# Relationships between priming and subsequent recognition memory

**DOI:** 10.1186/2193-1801-3-546

**Published:** 2014-09-22

**Authors:** Kiyofumi Miyoshi, Takehiro Minamoto, Hiroshi Ashida

**Affiliations:** Graduate School of Letters, Kyoto University, Sakyo, Kyoto, 6068501 Japan; Graduate School of Human Sciences, Osaka University, Suita, Osaka, Japan

**Keywords:** Priming, Implicit memory, Explicit memory, Recognition memory, Reaction time

## Abstract

A discrepancy exists among previous studies regarding whether priming and subsequent recognition memory are positively or negatively correlated. We consider that the difference in recognition memory measures used in these studies accounts for the discrepancy. To examine this, we introduced three different recognition measures and reexamined the relationship between priming and subsequent recognition. Participants learned stimulus words in the first encoding block while performing an abstract/concrete decision task. In the second encoding block, a priming test was conducted, followed by a surprise recognition memory test. Results showed that the hit rate and hit rate (pHit)–false-alarm rate (pFA) positively correlated with priming. However, the difference between hit rates for the twice- and once-encoded stimuli, which can reflect the representations acquired at the second exposure in particular, did not significantly correlate with priming. These results suggest that priming and subsequent recognition relate positively because of the common representations acquired at the initial encoding. Furthermore, the present results are consistent with a previous study that failed to reproduce the negative correlation between priming and subsequent recognition.

**Electronic supplementary material:**

The online version of this article (doi:10.1186/2193-1801-3-546) contains supplementary material, which is available to authorized users.

## Introduction

Human memory is not unitary but comprises multiple systems. Several studies have attempted to distinguish and explain the unique properties of these systems (for a review, see Cabeza and Moscovitch 2013). A fundamental topic in memory research is the distinction between implicit and explicit memory (Schacter and Tulving 1994). Implicit memory refers to the retrieval of pre-acquired representations without conscious awareness of memory (Tulving and Schacter 1990). Priming is one of the most researched implicit memory phenomena, conventional indicators of which are facilitated responses during tasks such as faster reaction time (RT) and greater accuracy. Explicit memory refers to conscious reflection of previously studied information or episodes, and it is often measured with a recognition or recall test. The present study investigates the relationship between these two types of memory.

Various brain and behavioral studies support the distinction between implicit and explicit memory (for reviews, see Schacter and Buckner 1998; Squire 2004; Tulving and Schacter 1990). Lesion studies have described a type of brain damage that impairs only explicit memory. For example, amnesic patients with damage in the medial temporal lobe exhibited deficits in explicit memory tests, but their performance in perceptual implicit memory tests was not affected (Blaxton 1992; Cermak et al. 1988; Graf et al. 1984; Hamann and Squire 1997). In addition, several neuroimaging studies have indicated that priming and explicit memory depend on different neural mechanisms (Paller et al. 2003; Schott et al. 2002, 2006; Spencer et al. 2009; Voss and Paller 2008). Furthermore, numerous behavioral studies have demonstrated that certain experimental manipulations affect priming and explicit memory differently (Tulving and Schacter 1990; Blaxton 1989; Moscovitch and Bentin 1993; Voss and Gonsalves 2010). For example, Voss and Gonsalves (2010) demonstrated that manipulating study duration had opposite effects on priming and explicit memory.

The abovementioned studies indicated that priming and explicit memory have different characteristics; however, they did not assert that these phenomena are completely independent. In fact, numerous studies have suggested that priming and explicit memory have common factors (for a review, see Dew and Cabeza 2011). For example, several studies have indicated that perceptual fluency caused by priming can underlie recognition memory (Jacoby and Whitehouse 1989; Rajaram 1993; Rajaram and Geraci 2000). Berry et al. (2008a, [b]) proposed a single-system computational model of priming and recognition memory and indicated that certain experimental dissociations between these phenomena can be explained without postulating independent sources of memory.

Several additional studies have directly assessed the relationship between priming and recognition memory (Gagnepain et al. 2008; Turk-Browne et al. 2006). In Turk-Browne et al. (2006), stimulus scenes were repeatedly presented, and the participants were required to report whether the scenes were indoor or outdoor. After this priming test, a surprise recognition memory test was conducted to assess the relationship between priming and subsequent recognition memory. The results revealed that subsequently recognized scenes associated with greater priming than those that were forgotten. Turk-Browne et al. (2006) suggests that priming and subsequent explicit memory relate positively because both of these two depend on the common representations acquired at the first exposure of the stimulus. Using a similar experimental design, Gagnepain et al. (2008) demonstrated that words associated with greater priming in an auditory lexical decision task were more accurately recognized in the subsequent recognition memory test. They suggest that priming and subsequent recognition memory relate positively because priming enhances the efficiency of explicit memory encoding at the second exposure of the stimulus. In sum, these studies suggest that priming and explicit memory are not completely independent and can correlate positively.

Contrary to these studies, however, Wagner et al. (2000) reported that priming correlated negatively to explicit memory. In their study, the participants incidentally encoded stimulus words in an abstract/concrete decision task and re-encoded them in a priming test following a 3-minute interval (short-lag condition) or a 25-hour interval (long-lag condition). After another 48-h interval, a surprise recognition test was conducted. They found (1) greater priming and lower recognition memory performance in the short-lag condition than in the long-lag condition, and (2) a negative correlation between priming magnitude and subsequent recognition performance in the long-lag condition across participants. Wagner et al. (2000) explained these negative relationships by proposing that priming hinders subsequent explicit memory by reducing encoding variability at the second exposure of the stimulus. More specifically, priming increases the likelihood of reprocessing only task-relevant features of the stimulus and decreases the likelihood of processing other features, which could lead to poor explicit memory.

These negative relationships observed in Wagner et al. (2000) have significant implications for understanding interactions between multiple forms of memory; therefore, they need further investigation. Stark et al. (2008) replicated the abovementioned result (1) of Wagner et al. (2000) and suggested that the lag between the first encoding and priming test affects priming and recognition memory in opposite directions; the lag suppresses priming but enhances recognition, which leads to the apparent negative relationships. However, they failed to replicate the result (2) of Wagner et al. (2000), which cannot be explained by the lag effect. Therefore, in the present study, we reexamined the across-participant negative correlation between priming and subsequent recognition.

In addition, it is of note that the mean magnitude of priming across participants was not significantly above zero in the long-lag condition of Wagner et al. (2000). This outcome could be problematic in assessing the relationship between priming and subsequent recognition memory. It was also uncertain whether their priming measures could indeed reflect priming. Therefore, in the present study, we excluded the 25-h interval between the first and second encoding to elicit stronger priming. One might point out that Wagner et al. (2000) have already shown no significant correlation in their short-lag condition across participants. However, they suggested that this lack of significant correlation may be because recognition memory performances obtained in that condition were restricted to a low range. Without the restriction, a strong negative correlation could have occurred in that condition, in which a strong priming effect was present. Therefore, to prevent such a floor effect, we shortened the interval between the second encoding and the surprise recognition test from 48-h to 24-h.

Another important objective of this study is to address the discrepancy among previous studies (Gagnepain et al. 2008; Turk-Browne et al. 2006; Wagner et al. 2000). As mentioned above, this discrepancy relates to whether priming and subsequent recognition memory are positively or negatively related. It is noteworthy that different measures of recognition memory were used in these studies as follows: hit rate (Turk-Browne et al. 2006), difference between hit and false alarm rates (pHit−pFA; Gagnepain et al. 2008), and difference between hit rates for the twice- and once-encoded stimuli (Wagner et al. 2000). Importantly, the measure employed by Wagner et al. (2000) may have a different property than the other standard recognition measures; as it is calculated by subtracting memory performance for once-encoded stimuli from that for twice-encoded stimuli, it especially reflects how efficiently participants encode stimuli at the second exposure, not at the first exposure (Wagner et al. 2000). Thus, we refer to it here as “learning efficiency at the second exposure”. We consider these different measures of recognition memory as the main reasons for the discrepancy among previous studies. Therefore, in the present study, we reexamined the relationship between priming and subsequent recognition using the three different recognition measures (hit rate, pHit−pFA, and learning efficiency at the second exposure).

## Materials and methods

### Participants

As participants for this study, 16 graduate and undergraduate students aged between 18 and 24 years (*M* = 21.9, *SD* = 1.73) volunteered, and they were paid according to the Kyoto University standard. Of the participants, 13 were male and 3 were female. All participants had normal color vision, and informed written consent was obtained from the participants before the experiment. All data were collected in accordance with the ethical principles of the American Psychological Association. This study was approved by the ethical committee of the Graduate School of Human Sciences at Osaka University.

### Materials

The stimuli comprised 150 Japanese nouns selected from the lexical database of imageability entitled “Lexical Properties of Japanese” (Amano and Kondo 1999). The items comprised two Chinese characters and had 3–4 morae. They included 75 abstract words (*imageability scores* of <3.0; *M* = 2.91, *SD* = 0.12) and 75 concrete words (*imageability scores* of >5.6; *M* = 5.86, *SD* = 0.25). In addition, 75 words were blue-colored and the other 75 were yellow-colored. The stimuli were semi-randomly assigned to three experimental conditions (novel, once-encoded, and twice-encoded) for each participant so that the number of abstract and concrete words would be the same across the conditions. A different color for each word was assigned for each participant regardless of the abstract/concrete category and it remained constant throughout the experiment. We introduced these colors for another purpose not described here. The effects of the color and abstract/concrete category on the results are summarized in Additional file 1. Stimuli were displayed on a dark background on a computer monitor (17″ Dell Ultra Scan P780) using the software Presentation (Neurobehavioral Systems). The distance between the monitor and participant was 50 cm.

### Procedure

#### First encoding block: first incidental learning

**Figure 1:**
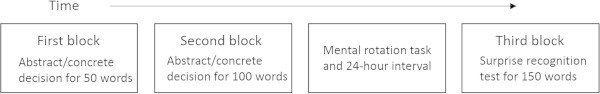
**Schematic diagram of experimental procedures.**

Figure 1 shows the schematic of the experimental procedure. In the first encoding block, 50 words were presented in a random order. As described above, half the words were concrete and the other half were abstract, and independently, half the words were yellow and the other half were blue. Each stimulus was presented for 1000 ms, and the participants were required to identify whether the stimulus was abstract or concrete as fast as possible by pressing the appropriate button on the keyboard. The intertrial intervals were 1000 ms, and unlike the long-lag condition of Wagner et al. (2000), the first encoding block was immediately followed by the second encoding block.

#### Second encoding block: priming test and second incidental learning

In the second encoding block, 50 novel words and 50 old words were presented in a random order, and the participants performed the abstract-concrete task as in the first encoding block. The participants were presented equal numbers of abstract and concrete words as well as equal numbers of blue and yellow words. As in the first encoding block, each word was presented for 1000 ms, and the intertrial intervals were 1000 ms. As the order of stimulus presentation was randomized, the possible minimum and maximal number of inter-repetition trials could be 0 and 148 respectively.

#### Surprise recognition memory test

The participants performed a mental rotation task as a distractor task for 3 min following the second encoding block, and the surprise recognition memory test was conducted after a 24-h interval. In this phase, 50 novel words, 50 once-encoded words, and 50 twice-encoded words were presented in a random order. The participants responded by identifying each word as old or new and reported their confidence (high or low) only when their response was old by pressing the appropriate button on the keyboard. There was no criterion for the confidence judgment. The words were presented with self-paced timing.

## Results

### Reaction time, priming magnitude, and recognition performance

Analyses included only trials with correct responses. RT data more than three standard deviations away from the mean were excluded as outliers for each participant. Table 1 lists the mean RTs and response accuracies in the encoding blocks. As in Wagner et al. (2000), priming magnitude was defined as the difference between the mean RTs for the novel and primed trials in the second encoding block for each participant. As expected, priming magnitude was significantly greater than 0 (*t* (15) = 7.51, *p* < .001). Table 2 lists the hit rates, false alarm rates, and pHit−pFA, which is widely used in memory research as the unbiased measure of recognition memory performance (See Snodgrass and Corwin 1988 for details).

**Table 1:** **Mean reaction times and response accuracies in each condition**

	First block	Novel in second block	Old in second block
Reaction time	698 (68)	705 (119)	647 (104)
Response accuracy	0.81 (0.11)	0.85 (0.07)	0.87 (0.06)

*SD* is shown in parenthesis.

**Table 2:** **Summary of recognition memory performance**

	High-confidence			Low-confidence			Overall		
	Hits	FAs	pHit-pFA	Hits	FAs	pHit-pFA	Hits	FAs	pHit-pFA
Once encoded	0.65	0.15	0.5	0.14	0.13	0.01	0.79	0.28	0.51
Twice encoded	0.84	0.15	0.69	0.09	0.13	-0.04	0.93	0.28	0.65
Learning efficiency	0.19			-0.05			0.14		

### Correlation between priming magnitude and pHit−pFA

**Figure 2:**
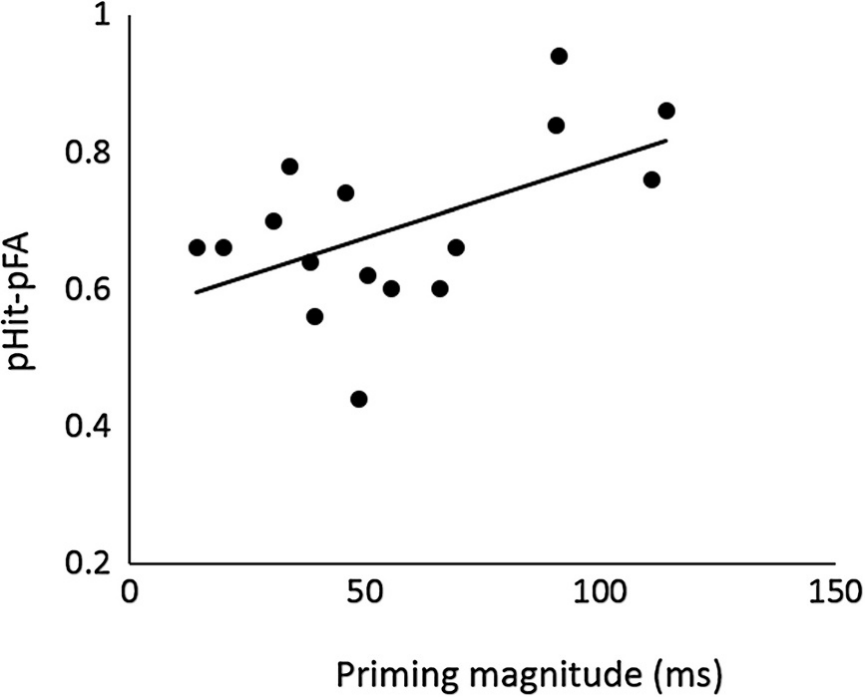
**Correlation between priming magnitude and high-confidence pHit−pFA for the twice-encoded stimuli.**

First, we investigated a correlation between priming magnitude and pHit−pFA across participants. Figure 2 shows a significant positive correlation between priming magnitude and high-confidence pHit−pFA for the twice-encoded stimuli (*r* = .54, *p* = .03); that is, the participants who exhibited greater priming performed better in the subsequent recognition memory test. The correlation between priming magnitude and overall (high- and low-confidence) pHit−pFA for the twice-encoded stimuli did not reach significance (*r* = .17, *p* = .53).

### Correlation between priming and learning efficiency at the second exposure

**Figure 3:**
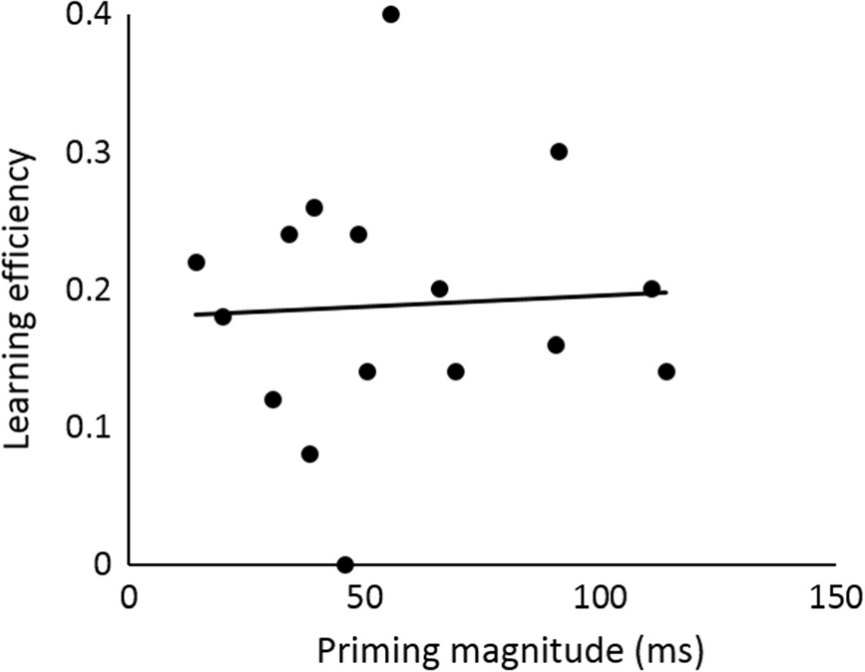
**Correlation between priming magnitude and high-confidence learning efficiency for the twice-encoded stimuli.**

Next, we conducted the same correlation analysis that Wagner et al. (2000) used. As their measure of recognition memory performance reflected primarily how well participants encoded the stimuli in the second encoding, we call it “learning efficiency at the second exposure” in the present study. Learning efficiency was defined as the difference between the hit rates for the twice- and once-encoded stimuli. We assessed the correlation between priming magnitude and learning efficiency at the second exposure across participants. When learning efficiency was calculated on the basis of only the high-confidence hit rate, there was no significant correlation between priming magnitude and learning efficiency (*r* = .05, *p* = .85) (Figure 3). Moreover, when learning efficiency was calculated on the basis of the overall hit rate, the correlation did not reach significance (*r* = −.29, *p* = .27); that is, the significant negative correlation observed in Wagner et al. (2000) was not replicated in the present study. These lines of correlational analyses suggest that the positive correlation shown in Figure 2 may have occurred because priming and subsequent recognition memory depended on the memory representations acquired in the first encoding, not because priming enhanced learning efficiency in the second encoding.

### Analysis of individual stimuli with the hit rate

**Figure 4:**
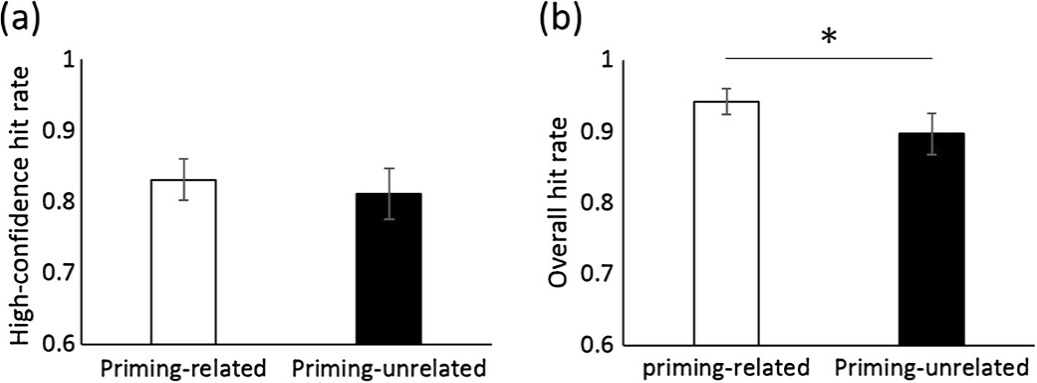
**Analysis of individual stimuli. (a)** The high-confidence hit rate for priming-related and priming-unrelated words; **(b)** The overall hit rate for priming-related and priming-unrelated words. Error bars indicate the standard error of the mean. *indicates *p* < .05.

Lastly, we conducted an intra-participant analysis of individual stimuli using the hit rate. Unlike correlation analysis across participants, this intra-participant analysis is not contaminated by a participant’s response biases even when using the hit rate not corrected by subtracting the false alarm rate. First, we calculated the individual amount of priming for the 50 words (RT in the first encoding block- RT in the second encoding block) for each participant. We then divided the words into two groups: priming-related words (words associated with a positive amount of priming) and priming-unrelated words (words not associated with a positive amount of priming). Paired t-tests indicated that the overall hit rate for priming-related words was significantly higher than that for priming-unrelated words (*t* (15) = 2.67, *p* = .02); however, there was no significant difference between the high-confidence hit rate for priming-related words and that for priming-unrelated words (*t* (15) = 0.88, *p* = .39) (Figure 4).

## Discussion

### Positive relationships between priming and subsequent recognition memory

In the present study, we re-examined the relationship between priming in the abstract/concrete decision task and subsequent recognition performance with a modified procedure based on Wagner et al. (2000). By excluding the interval between the first and second encoding blocks, we observed a significant priming effect, which was not found in the long-lag condition in Wagner et al. (2000). The results demonstrated positive relationships between priming and subsequent recognition indicated by the hit rate and pHit−pFA. However, we found no significant correlation between priming and learning efficiency at the second exposure.

Gagnepain et al. (2008) suggested that priming enhances the formation of explicit memory at the second encoding of the stimulus; however, the present results indicate that priming might not directly enhance efficiency of explicit memory encoding at the second exposure. Instead, our results suggest that priming and subsequent recognition relate positively on the basis of the common representations acquired at the first exposure. Turk-Browne et al. (2006) suggested that attention at the first exposure benefits both priming and explicit memory, and several other studies have suggested that both explicit memory and priming can be subject to attentional modulation (Mulligan 1998; Naccache et al. 2002; Spataro et al. 2011). Thus, the positive correlations observed here might occur because priming and recognition memory depend on common encoding factors and representations acquired in the initial study. The participants who paid more attention to the stimulus at the first encoding may have acquired stronger memory representations and have performed well in both the priming and recognition memory tests. Likewise, the words attracting more attention may have been more efficiently represented in memory and better retrieved in both tests. In fact, the results of Gagnepain et al. (2008) can also be explained by this account.

Wang et al. (2010) reported that both explicit memory and conceptual priming can depend on the medial temporal lobe. Moreover, numerous studies have reported similarities between conceptual priming and familiarity-based recognition memory (Dew and Cabeza 2011). Thus, these similarities may enable the positive correlation between priming and subsequent recognition memory observed in the present study.

### Lack of significant correlation between priming and learning efficiency

Similar to Stark et al. (2008), we did not replicate the negative correlation across participants observed in the long-lag condition of Wagner et al. (2000). As mentioned before, there was no significant priming effect in the long-lag condition of Wagner et al. (2000). This could indicate that the variability across participants in the priming measure in this particular condition indexes something other than variability in priming. According to this idea, the negative correlation observed across participants in Wagner et al. (2000) might have arisen merely because the participants taking more time to process old stimuli in the second encoding exhibited greater learning efficiency at the second exposure. However, it is still possible that methodological differences between our study and theirs (e.g., difference in time lag) are responsible for the discrepancy in results.

Alternatively, Xue et al. (2010, 2011) suggested that priming-associated neural deactivation (repetition suppression) during encoding impairs subsequent explicit memory. However, they provided no behavioral measure of priming to demonstrate its relationship with repetition suppression. In addition, it is suggested that neural repetition suppression and behavioral priming are not always associated (Sayres and Grill-Spector 2006). More direct and conclusive evidence is required to determine whether there is a negative influence of priming on the efficiency of explicit memory encoding.

### Implicit memory processes in multiple forms of recognition memory

In accordance with previous studies (Rajaram and Geraci 2000; Sheldon and Moscovitch 2010; Voss et al. 2008), the present study suggests that implicit memory processes are involved in the recognition memory test. Several studies have reported that presenting a masked prime immediately before the target stimulus in the recognition test increased the occurrence of familiarity-based recognition (Rajaram 1993; Rajaram and Geraci 2000). In addition, several studies have suggested that recognition memory could be based on implicit memory processes even when participants are unaware of memory retrieval, a phenomenon referred to as “implicit recognition” (Miyoshi and Ashida 2014; Voss et al. 2008; Voss and Paller 2009). To summarize, various implicit memory processes are involved in different forms of recognition memory. Careful attention should be given to the influence of these implicit processes in various types of memory tests.

## Electronic supplementary material

Additional file 1: **Effects of the color and abstract/concrete category on results.** (DOCX 17 KB)
