# Supplementary material for: Relationships between priming and subsequent recognition memory
Source: Springerplus. 2014 Sep 22;3:546. doi: 10.1186/2193-1801-3-546 (PMC4182323; doi:10.1186/2193-1801-3-546)
Supplement: Supplementary file 1 — Additional file 1: Effects of the color and abstract/concrete category on results. (DOCX 17 KB) [file 40064_2014_1252_MOESM1_ESM.docx]

1. **Effect of color and abstract/concrete category on priming magnitude**

A two-way repeated measures ANOVA was conducted on priming magnitude with stimulus color and abstract/concrete category as within-participant factors. There was no significant main effect of stimulus color (*F* (1, 15) = 0.39, *p* = .54) or abstract/concrete category (*F* (1, 15) = 2.47, *p* = .14), nor was there a significant interaction (*F* (1, 15) = 0.30, *p* = .59).

**Table S1. Priming magnitude for each stimulus category.**

|  | Priming magnitude (ms) |
| --- | --- |
| Blue concrete | 38 (100) |
| Blue abstract | 66 (61) |
| Yellow concrete | 52 (49) |
| Yellow abstract | 61 (90) |

*SD* is shown in parenthesis.

1. **Effect of color and abstract/concrete category on high-confidence recognition performance for twice-encoded stimuli**

First, we conducted a two-way repeated measures ANOVA on the high-confidence hit rate with stimulus color and abstract/concrete category as within-participant factors. There was no significant main effect of stimulus color (*F* (1, 15) = 3.60, *p* = .08) or abstract/concrete category (*F* (1, 15) = 2.57, *p* = .12), nor was there a significant interaction (*F* (1, 15) = 0.18, *p* = .68).

Second, we conducted a two-way repeated measures ANOVA on the high-confidence pHit-pFA. There was a significant main effect of stimulus color (*F* (1, 15) = 8.35, *p* = .01) but no significant main effect of abstract/concrete category (*F* (1, 15) = 3.80, *p* = .07). There was no significant interaction (*F* (1, 15) = 0.60, *p* = .45). Although the significant main effect of stimulus color was not anticipated, it does not undermine the main results of the correlational analysis across participants because all participants encoded the same number of yellow and blue stimuli.

Third, we conducted a two-way repeated measures ANOVA on the high-confidence learning efficiency. There was no significant main effect of stimulus color (*F* (1, 15) = 0.01, *p* = .91) or abstract/concrete category (*F* (1, 15) = 0.71, *p* = .41), nor was there a significant interaction (*F* (1, 15) = 0.18, *p* = .67).

**Table S2. High-confidence recognition memory performance for each stimulus category.**

|  | Hit rate | pHit-pFA | Learning efficiency |
| --- | --- | --- | --- |
| Blue concrete | 0.83 (0.17) | 0.70 (0.17) | 0.21 (0.16) |
| Blue abstract | 0.89 (0.14) | 0.77 (0.21) | 0.16 (0.18) |
| Yellow concrete | 0.78 (0.17) | 0.59 (0.18) | 0.19 (0.16) |
| Yellow abstract | 0.86 (0.13) | 0.70 (0.15) | 0.18 (0.14) |

*SD* is shown in parenthesis.

1. **Effect of color and abstract/concrete category on overall recognition performance for twice-encoded stimuli**

We conducted a two-way repeated measures ANOVA on the overall hit rate with stimulus color and abstract/concrete category as within-participant factors. There was no significant main effect of stimulus color (*F* (1, 15) = 2.37, *p* = .14) or abstract/concrete category (*F* (1, 15) = 2.14, *p* = .16), nor was there a significant interaction (*F* (1, 15) = 0.07, *p* = .80).

Next, we conducted a two-way repeated measures ANOVA on the overall pHit-pFA. There was a significant main effect of stimulus color (*F* (1, 15) = 6.40, *p* = .02) but no significant main effect of abstract/concrete category (*F* (1, 15) = 4.04, *p* = .06). There was no significant interaction (*F* (1, 15) = 0.59, *p* = .45). As mentioned in the previous section, the significant main effect of stimulus color does not undermine the main results of this study.

Lastly, we conducted a two-way repeated measures ANOVA on the overall learning efficiency. There was no significant main effect of stimulus color (*F* (1, 15) = 0.01, *p* = .94) or abstract/concrete category (*F* (1, 15) = 0.67, *p* = .43), nor was there a significant interaction (*F* (1, 15) = 0.36, *p* = .56).

**Table S3. Overall recognition memory performance for each stimulus category.**

|  | Hit rate | pHit-pFA | Learning efficiency |
| --- | --- | --- | --- |
| Blue concrete | 0.92 (0.11) | 0.65 (0.19) | 0.16 (0.15) |
| Blue abstract | 0.97 (0.05) | 0.72 (0.16) | 0.12 (0.09) |
| Yellow concrete | 0.90 (0.16) | 0.55 (0.20) | 0.14 (0.13) |
| Yellow abstract | 0.95 (0.08) | 0.68 (0.11) | 0.14 (0.13) |

*SD* is shown in parenthesis.
